# Forecasting Multiple Groundwater Time Series with Local and Global Deep Learning Networks

**DOI:** 10.3390/ijerph19095091

**Published:** 2022-04-22

**Authors:** Stephanie R. Clark, Dan Pagendam, Louise Ryan

**Affiliations:** 1 Commonwealth Scientific and Industrial Research Organisation, Canberra, ACT 2601, Australia; stephanie.clark@csiro.au (S.R.C.); dan.pagendam@data61.csiro.au (D.P.); 2 School of Mathematical and Physical Sciences, University of Technology Sydney, Sydney, NSW 2170, Australia

**Keywords:** time series, recurrent neural networks, long short-term memory (LSTM), self-organising map (SOM), DeepAR, groundwater

## Abstract

Time series data from environmental monitoring stations are often analysed with machine learning methods on an individual basis, however recent advances in the machine learning field point to the advantages of incorporating multiple related time series from the same monitoring network within a ‘global’ model. This approach provides the opportunity for larger training data sets, allows information to be shared across the network, leading to greater generalisability, and can overcome issues encountered in the individual time series, such as small datasets or missing data. We present a case study involving the analysis of 165 time series from groundwater monitoring wells in the Namoi region of Australia. Analyses of the multiple time series using a variety of different aggregations are compared and contrasted (with single time series, subsets, and all of the time series together), using variations of the multilayer perceptron (MLP), self-organizing map (SOM), long short-term memory (LSTM), and a recently developed LSTM extension (DeepAR) that incorporates autoregressive terms and handles multiple time series. The benefits, in terms of prediction performance, of these various approaches are investigated, and challenges such as differing measurement frequencies and variations in temporal patterns between the time series are discussed. We conclude with some discussion regarding recommendations and opportunities associated with using networks of environmental data to help inform future resource-related decision making.

## 1. Introduction

Groundwater is an essential source of freshwater for much of the world’s population. In many places, however, available groundwater resources are under stress due to increasing anthropogenic influences and demand, as well as a changing climate. The appropriate monitoring and modelling of these groundwater systems are critical to enabling management decisions that will lead to a sustainable future.

Classical groundwater modelling approaches use mathematical models consisting of complex systems of differential equations to represent the physical processes known to contribute to groundwater levels. However, these models require substantial assumptions and are typically subject to considerable uncertainty. In particular, accurately characterizing the hydrogeological properties of an area with a physically based model requires extensive expert hydrogeological knowledge and many assumptions about the nature of underground structures and the mechanisms involved in groundwater recharge. Groundwater systems are often complex, with water levels depending on many static and time-varying influences, including long- and short-term climate conditions, vegetation, land use, soil permeability, hydraulic conductivities, subsurface geological structures, aquifer size and connectivity, extraction patterns, recharge from local rivers and lakes, overland flooding, and irrigation activities. Gathering relevant information on each of these variables is usually difficult, time-consuming, and expensive. Building models for areas where there is not access to this information require the incorporation of many assumptions. In areas where subsurface information is available, the climate, soil, and vegetation characteristics (e.g., transpiration rate, cover, root systems, etc.) continually evolve over time, contributing temporal changes to groundwater recharge mechanisms and rendering these systems even more difficult to understand and represent through explicitly defined mathematical relationships. In general, the more realistic a physically based model is, the more data and assumptions will be required. See [1] for a recent textbook on the topic.

To preclude this need for extensive knowledge about the subsurface systems, groundwater modellers are increasingly turning to data-driven approaches that use statistical modelling and machine learning to make predictions. In recent years, these data-driven approaches have gravitated towards the use of neural networks and deep learning algorithms (see [2,3,4]). The growing popularity of neural networks in hydrogeological prediction is due to their ability to extract features, represent relationships, and make predictions on complex systems without requiring detailed knowledge about the physical bases of the underlying system. This in turn allows patterns to emerge without requiring strong assumptions about the unknown factors and linkages [5,6,7,8]. Depending on data availability, these models also have the potential to incorporate the impact of anthropogenic influences on the hydrogeologic system without explicitly quantifying the relationships in advance. Keeping up with the increasing capacity to collect hydrologic data, deep learning systems provide a means of efficiently processing these large data sets [6]. Big data techniques (e.g., incorporating global climate models, remote sensing, citizen science, etc.) have been shown to benefit sustainable groundwater management by overcoming a lack of relevant data at the local scale [9]. There is increasing awareness of the amount of information that can be extracted with data-driven models and acknowledgments that our ability to make predictions from data is improving at a greater rate than our ability to make predictions from hydrologic theory [8]. The rapid expansion of neural network methodology from the machine learning field is continually adding new ideas to existing groundwater modelling possibilities.

**Figure 1:**
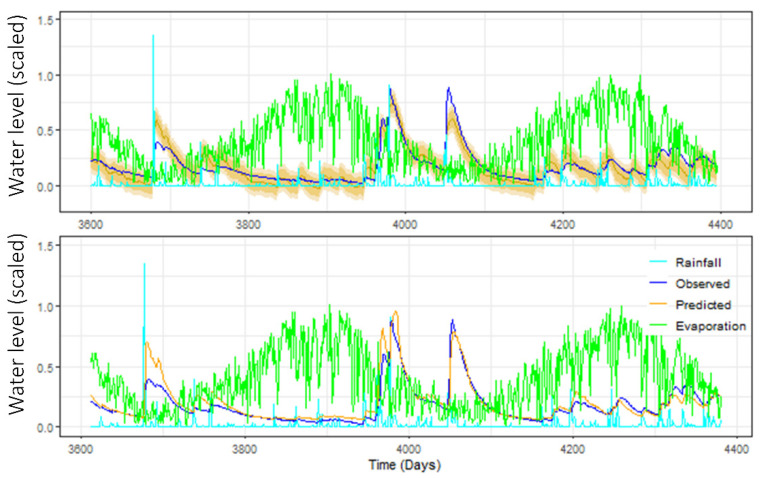
Observed (dark blue) and predicted (orange) groundwater levels from a classical time series model (upper plot) and a neural network model (lower plot). The classical prediction includes 80 and 95% prediction intervals, also shaded in orange. Adapted with permission from [10]. Copyright 2020 John Wiley & Sons.

In a recent project sponsored by the NSW Department of Planning and Environment (DPIE) in New South Wales (NSW), Australia, we compared machine learning approaches based on neural network models with classic time series methods to model the level of groundwater in an aquifer in the Richmond River catchment, in the northeast part of the state [10]. We found that, while each approach has some unique advantages and disadvantages, both did a remarkably good job at capturing changing patterns over time. The results of this project are depicted in Figure 1, showing groundwater levels (dark blue), along with rainfall levels (light blue), evaporation (green) and predictions (orange), based on classic autoregressive integrated moving average (ARIMA) modelling (top panel) and a neural network model (bottom panel). Neither approach gives a perfect prediction, but both do very well in terms of capturing the key features of how the aquifer levels changed over time. See [10] for more detail.

In a follow-up project for DPIE, we set out to apply the same analyses to data from a different catchment area, the Namoi River in the north-west part of the state. However, the results were not nearly as good. It became quickly apparent that, in contrast to the Richmond catchment, the Namoi analysis was significantly more complex due to being an area of relatively low rainfall combined with high groundwater extractions to meet the demands of intensive agriculture and mining industries. It was clear that effective modelling needed to draw on a more extensive range of potential predictors, including data related to extractions, as well as river flow rates (which were also indirect indicators of dam releases during dry periods). Additionally, the data sets from each monitoring bore were relatively small in terms of the number of observations, and exhibited large proportions of missing data, impeding the application of individual neural network time series models to each well. We decided that it would make sense to work with a larger, richer dataset that combined data from multiple aquifers in the catchment, to share information across the region and not simply analyse on a bore-by-bore basis, as had been successful for the Richmond River catchment. The analysis was complicated by high levels of spatiotemporal variability between the individual groundwater time series measured in the region. This present paper is a detailed case study describing our efforts to undertake this analysis based on multiple, inter-related time series corresponding to 165 groundwater monitoring bores in the Namoi River catchment. 

There is currently growing interest from the machine learning community around the use of global, rather than local, models for time series analysis [11,12,13]. Local (or individual) time series prediction models are trained on a single time series, usually under the assumption that each time series results from a unique data generating process. On the other hand, global models are trained on data from multiple time series simultaneously; the data is aggregated into a single data set and a single model is produced. Another variation, known as partitioned models, falls between local and global, working on a subset of related time series. Global or partitioned models by nature have access to larger sample sizes than local models for the same data, and it is well known that machine learning models work best when there is a large volume of input data. While individual models representing a complex system with relatively few data points may tend to overfit, global models have the advantage of borrowing strength from a larger pool of data and are therefore less likely to overfit, potentially leading to better generalisation to new data. 

It has been shown [11] that global models do indeed generalise better than individual models to data that have not been seen during the training process, and that ‘long memory patterns and related effects’ that would require manual introduction into local models are better able to be learned automatically by global models. Moreover, Ref. [12] found that an LSTM model (long short-term memory [14]) trained over multiple time series performed better than univariate methods if the time series are similar. The authors discuss the capabilities of neural networks to operate as universal function approximators that ‘make them ideal for exploiting information across many time series’. They used a global LSTM to make predictions on time series that had been clustered based on the similarity of their features, and determined that grouping the time series by similarity increased prediction capabilities. Ref. [13] concluded that the combination of recurrent neural networks such as LSTM with the leveraging of cross-series information through global models led to benefits in forecasting accuracy. Ref. [15] proposed a global time series model, implemented with publically available software called DeepAR, that incorporated the LSTM structure along with the inclusion of lagged outcomes as additional predictors to produce probabilistic forecasts on multiple time series at once. 

In a hydrological context, Ref. [8] describes how traditional models perform best when calibrated on individual basins, but deep learning works best when trained on multiple catchments, as shown by [16]. In this study, the authors train a single LSTM on runoff time series from over 500 basins and produce better predictions than with various benchmark hydrological models calibrated individually by catchment. Ref. [17] demonstrated the power of a global model to forecast groundwater levels across the state of Victoria in Australia. Ref. [5] reviews machine learning applications in hydrology to date, finding that most are studies of small datasets at individual sites that are not transferable to other locations. The authors suggest that the future lies in multitask learning, where machine learning tasks have several target variables. 

The main purpose of this paper is to present a case study, comparing and evaluating the use of local, global and partitioned time series models on the time series from the 165 wells from the Namoi River catchment. Located in the same region, the time series have been created by similar, though not exactly the same, data-generating processes, indicating that it may be useful to share some information across the system. Climatic conditions of rainfall and evapotranspiration are closely related for all wells, but subsurface conditions affecting recharge rates, such as soil permeability, aquifer depth, and hydraulic connectivity, will affect each time series differently. We explore whether these system differences indicate that a separate model should be made for each time series, or if combining the time series is beneficial for prediction performance. If so, should they be subsetted in a meaningful way (i.e., partitioned based on the similarities of their temporal patterns) or simply all combined together? The benefits of modelling this set of related time series with the possible approaches (individually, partitioned, or agglomerated into a single global model) are investigated and quantified here. 

To the best of our knowledge, this study represents the first application of the DeepAR technology in the context of hydrogeology. We also provide a general discussion of the benefits and limitations of the application of various contemporary machine learning multiple-time series methods to real-world environmental monitoring data. A key insight is that, while machine learning strategies such as DeepAR can do well in terms of short-term predictions, long term predictions require models where key drivers have been identified, measured well, and incorporated into the modelling process. 

Section 2 provides more detail about the Namoi River catchment and the data available for our analysis. An overview of the various methods that we have considered is given in Section 3. Section 4 describes the results of applying these methods to the Namoi data. In Section 5 and Section 6, the results are discussed, and some final conclusions are drawn, including some discussion about potentially useful extensions of the modelling framework. 

## 2. Data and Study Area

**Figure 2:**
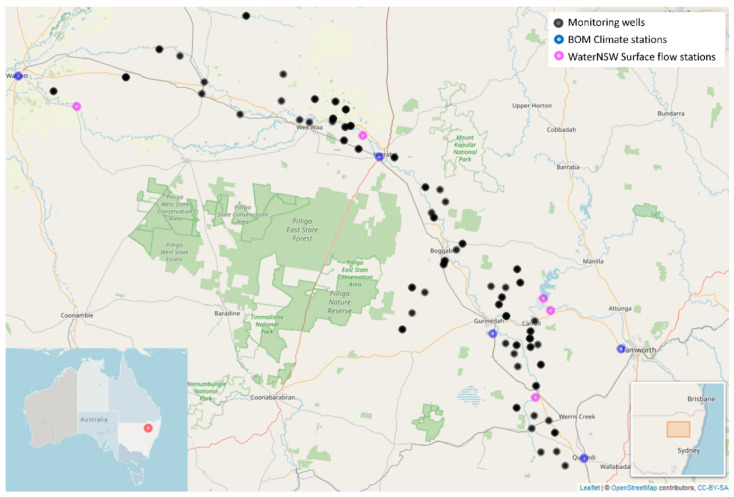
Monitoring sites and environmental monitoring stations used in this study. Adapted with permission from ref [18]. Copyright 2022 Elsevier.

In this paper, we focus on the analysis of 165 groundwater level time series from 70 different monitoring locations across the Namoi River catchment in northern NSW, located just to the west of the Great Dividing Range. Multiple monitoring bores, or wells, are often established at the same location, in order to allow access to aquifers at different depths, hence the greater number of time series than sites. The locations of the monitoring sites and environmental monitoring locations are shown in Figure 2. The study period is 1 January 1974 to 31 December 2018. 

**Figure 3:**
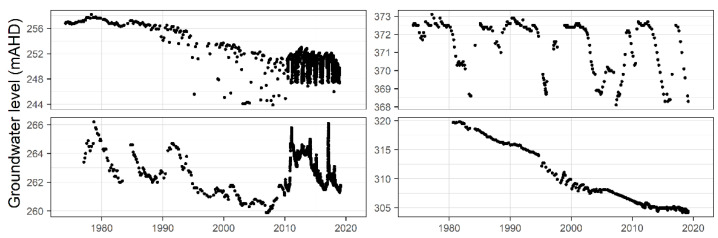
A sample of the 165 groundwater time series that make up the data set. Varying temporal patterns and record lengths are evident among the time series.

**Figure 4:**
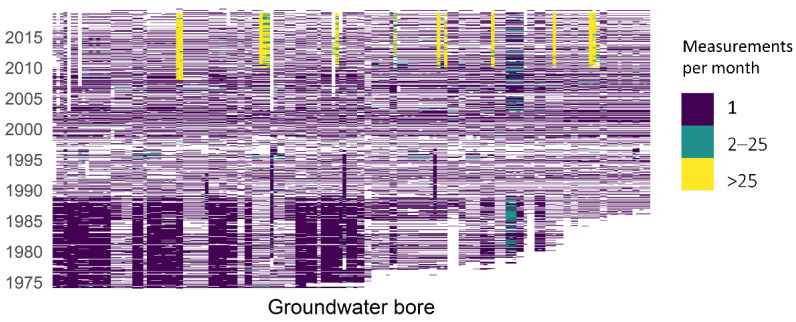
Measurement frequency at the 165 groundwater bores in the data set. Bores are represented by columns arranged from left to right by site number, which roughly follows date of station commissioning. For each bore, the months of station operation are represented vertically. Yellow indicates months with >25 measurements (i.e., daily), identifying the period of telemetry for some of the bores. White indicates no measurements.

Groundwater level monitoring data have been provided by the water division of the NSW Department of Planning, Industry and Environment (DPIE). These data are also publicly available for download from a website maintained by WaterNSW [19], a state-owned corporation established under 2014 legislation to manage and oversee NSW water resources. WaterNSW owns and operates the largest surface and groundwater monitoring system in the southern hemisphere. The recorded groundwater measurements show high variability from well to well, and many of the time series contain strong temporal patterns, as can be seen in the sample of four sets of measurements shown in Figure 3. The time series are of differing lengths due to different dates of station commissioning and/or decommissioning. They are characterised by sporadic measurement frequencies and high levels of missing data. At the beginning of the records, the measurements were made manually on a 2–3-month rotation. In recent years, a few stations have had automatic telemetry equipment installed and are recording regular daily measurements. There was a total of 11 bores in this study with automatic telemeters installed. Figure 4 gives an indication of the measurement frequency at each bore and gaps in the data set.

Rainfall and evapotranspiration measurements were obtained at a daily resolution from the SILO database [20], constructed and maintained by the Australian Bureau of Meteorology (BOM) and comprising observed values, along with infilled missing values. The specific SILO evapotranspiration variable used is ‘Penman-Monteith reference evapotranspiration (FAO56)’. Rainfall and evapotranspiration data from five climate stations across the Namoi catchment are used in this study: Walgett Council Depot (station 52026), Narrabri West Post Office (station 53030), Gunnedah Resource Centre (station 55024), Tamworth (station 55054), and Quirindi Post Office (station 55049). The rainfall data vary greatly across the region, while evapotranspiration follows a similar pattern for these stations.

River discharge data were downloaded directly from the WaterNSW website at daily resolution for the following stations: Goangra (station 419026, upstream of Walgett), Mollee (station 419039, downstream of Mollee weir between Narrabri and Weewaa), downstream of Keepit Dam (station 419007), the Peel River at Carroll Gap (station 419006), and the Mooki River at Breeza (station 419027). Patterns of measured streamflow are complicated in this region, influenced by a combination of natural phenomena and human interventions. Dam outflow rates may be increased during periods of low rainfall due to intentional dam releases, leading to elevated downstream flow. Of course, streamflow also fluctuates naturally during periods of high and low rainfall. 

Extraction (groundwater pumping) data were provided by DPIE in the form of annual extracted volumes (ML/year) at locations specified by latitude and longitude. These records begin in 1967 for some of the wells, and in 1985 for others. Due to a lack of recorded data, in this study, we assume that wells with no records before 1985 did not have pumping occurring. In actuality, the lack of reliability in the recorded extractions data may be a source of uncertainty that impacts the reliability of the fitted models, and we discuss this issue further in our discussion section. To integrate this annual lump-sum data with the daily environmental measurements, extractions have been set at a constant value throughout the year. As discussed presently, the inclusion of a day- or month-within-year variable in our models allows the flexibility for the neural networks to create interactions that help explain annual fluctuations.

**Figure 5:**
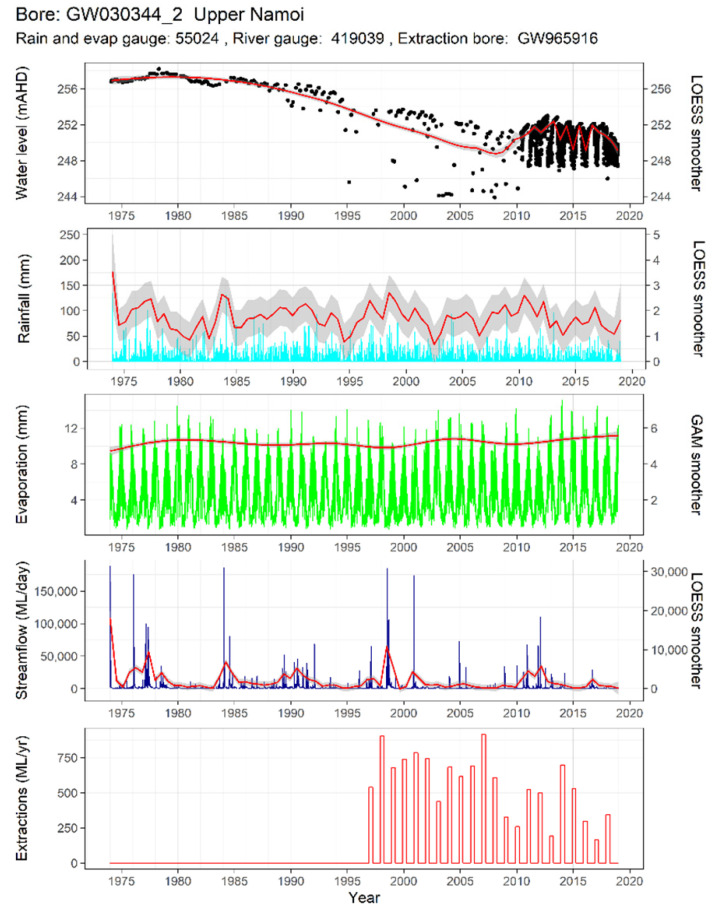
Groundwater hydrograph and some associated predictors for a single well (GW030344_2). Non-parametric smoothers have been added (red lines) to make any long-term trends more evident.

Figure 5 shows water level data from one groundwater monitoring well, GW030344_2, with some of the predictor time series that will be used to model the water levels. Only data from a single gauge of each of the predictors are shown, though in the models, there are multiple inputs of each type of predictor (i.e., many extraction bores). Note that this is one of the wells where an automatic telemeter had been installed around 2010. 

The study region forms part of the Murray–Darling basin and is a highly productive agricultural area sustained by large volumes of groundwater extractions. As the subsurface characteristics of this area are complicated and not yet fully defined, classical, process-based hydrogeological modelling is difficult to apply. It is known that the groundwater system consists of multiple layers of aquifers, with unmeasured lateral through-flow and vertical leakage occurring between the shallow and deep aquifers. The surface water and groundwater systems are closely connected, meaning that groundwater depletions due to extractions may be masked by incoming surface water. Large amounts of water that are extracted from deep aquifers end up finding their way into shallow aquifers via irrigation. Surface waterways are substantially regulated by dams and weirs, providing a disconnection between rainfall events and groundwater recharge. This relationship is complicated further in that periods of low rainfall can lead to high extractions that in turn deplete groundwater, and yet low rainfall can also lead to dam releases that lead to recharge through the streambeds. The amount of rainfall varies greatly between the east and west of the region, and the response of groundwater levels to precipitation can vary between areas even within the same aquifer, due to differences in soil permeability. Geological fault lines disconnect subsurface hydraulic characteristics. The complex hydrogeology of the region, along with the extensive monitoring network in place, makes it very natural to consider empirically based approaches. 

## 3. Methods

### 3.1. Overview of Our Approach

The consideration of Figure 2 naturally leads to the question of whether it would be useful to consider some type of classic spatio-temporal model (see [21]) which is designed for settings where the data to be modelled are indexed in both space and time. However, such methods are not a natural fit in the present context for several reasons. First of all, the wells tend to lie in long narrow ribbons, following the courses of the various rivers and waterways in the region. This kind of spatial distribution can lead to instabilities in model fitting since it means that there is a long-tailed distribution of spatial distances in the dataset. More importantly, the traditional approach to spatio-temporal modelling requires the specification of a spatial correlation structure. The typical assumption is that the correlation between any two pairs of data points is a function of how far apart they are. In the context of groundwater modelling, such an assumption would be entirely inappropriate. The correlation between any two points is likely to be a complex function, reflecting the nature of the latent underground structures. Two wells that are quite close to each other in terms of their location on a map could have a low correlation with each other if they each are tapping into different aquifers (e.g., one taps into a deep aquifer, one a shallow aquifer). Similarly, two wells could be far apart, yet correlated because they each tap into the same aquifer. We briefly explored the use of spatio-temporal models using the R package spTimer, but it did not yield helpful results, and seemed computationally unstable for our data. For these reasons, spatio-temporal methods were not explored further, and instead we focused on the use of time series methodologies. 

We begin by attempting to model the time series with ‘local’, or individual, neural network models using the basic multi-layer perceptron (MLP) architecture. These local models are produced by fitting a separate model to each time series, resulting in a number of models equal to the number of monitoring wells. This method is based on the assumption that each time series is the result of a different data-generating process, and separate models are warranted to represent the individual processes. Because large datasets are needed for training neural networks, this local modelling step is restricted to those wells with more than 2000 observations, which is the small subset of telemetered wells in the region.

Next, for a potentially more efficient use of the data than could be obtained from the individual models, and to incorporate data from non-telemetered wells as well, we turn to an exploration of multiple time series models. Multiple time series models have the benefits of larger datasets for model training, and the sharing of pattern information between the time series from some or all of the wells. These ‘multi-well’ models can be either fit on the entire set of time series (‘global’ models) or on subsets of the time series (‘partitioned’ models). The input data for the multi-well models consist of one large, stacked set of data from multiple wells, along with a new predictor variable that indicates from which well each measurement in the dataset was taken. Due to the process of pooling the data, there is the potential for greater generalisation and a lower prospect of over-fitting with these multiple time series models than with local models. 

We investigate three versions of multiple time series models with these groundwater monitoring data:

(1) the time series are first clustered and then a recurrent neural network model specifically designed for the time series (the LSTM, or long short-term memory model, [14]) is applied to each cluster,
(2) a global, standard neural network (MLP) is created using all the time series, and
(3) a global, recurrent neural network (LSTM-based) is created using all the time series with the DeepAR algorithm [15]. As discussed in more detail in Section 3.3.4, DeepAR is a global time series prediction algorithm that combines elements of LSTM modelling with classical time series autocorrelation structures, with the added capability of producing probabilistic predictions.

The first approach is a partitioned model, where a model is produced to represent a subset of similar time series from the overall group, as determined by a clustering measure. The latter two approaches are global models, in which a single model is created to represent the data from all of the time series. These algorithms will each be described in detail below.

### 3.2. Single Well Analyses

#### 3.2.1. Classical Statistical Formulation

The foundational concept underlying time series modelling is that future values of the time series can be more accurately predicted by exploiting the correlation structure inherent in the data. The term ‘autocorrelation’ refers to a special kind of correlation that arises in the context of time series. The idea is that observations measured more closely together in time are likely to be more similar than observations measured farther apart. We are interested in a subclass of these models where, along with the outcome *Y_t_*, we have a set of predictors (or features). denoted *X_t_*, that can be used in constructing a prediction model. For our project, we consider dynamic regression models which take the following form:

(1) $$Y_{t}=BX_{t}+e_{t}$$

for time series {*Y*_1_, *Y*_2_, …, *Y_T_*} where *Y_t_* represents the well water level measured at time *t*, *B* is a vector of regression coefficients to be estimated and *e_t_* is an error term that can incorporate additional autocorrelation structure, if needed. In practice, the general philosophy is that the autocorrelation observable in a set of time series data can often be explained by measuring the right features. The best and most reliable predictions, particularly for the longer term, will result from models that incorporate a rich and relevant set of additional features. In [10], results obtained through this type of classic statistical time series modelling were compared with those obtained through the application of machine learning strategies.

#### 3.2.2. Multi-Layer Perceptron (MLP)

**Figure 6:**
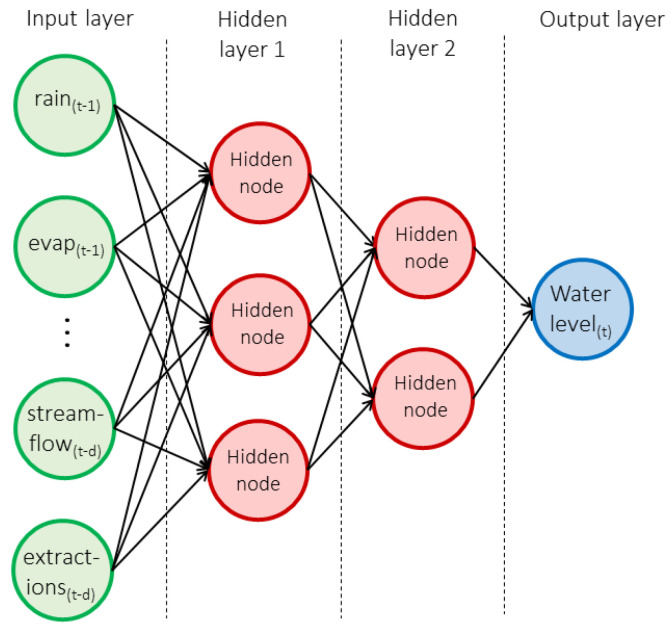
Two-layer MLP, with 3 nodes on the first hidden layer and 2 nodes on the second.

The multi-layer perceptron (MLP) is the simplest example of a supervised neural network. Like the classical statistical model, an MLP takes a set of input variables (*X*, also called predictors or features) and uses these to predict an outcome, *Y*. However, unlike the model in Equation (1) above, the MLP assumes the existence of a set of interconnected hidden layers, each of which is a function of nodes lower in the hierarchy. Data enter the network at the input layer, where each variable is represented by an input node, and then flows in one direction through the network from the input layer to the output layer. It is through the inclusion of these hidden nodes that the MLP can discover important interactions between the input variables that will be used to predict an outcome. Figure 6 illustrates an MLP with two hidden layers that predicts water level at a given time based on a set of predictors measured at past times. 

In the MLP, each connection between nodes has an associated weight, or parameter, to be estimated. As the data pass through the network at each iteration, a bias is added to the summed, weighted inputs before the data are run through an activation function. The activation function provides a nonlinear mapping between the inputs and outputs of each node. An MLP with one hidden layer can be represented as:

(2) $$y=g_{\;}\left(W_{ho}f\left(W_{ih}x+b_{h}\right)+b_{o}\right)$$

where x is a vector of input variables, $W_{ih}$ and $W_{ho}$ are matrices of weights (between the input and hidden layer, and hidden layer and output layer respectively), $b_{h}$ and $b_{o}$ are bias terms (model intercepts in statistical terms), f(·) is the activation at the hidden layer, and g(·) is the activation at the output layer. Ref. [10] provides a more detailed discussion of the MLP, as well as relevant references. 

The neural network training process consists of iteratively updating the weights and biases by feeding labelled input/output data through the model and comparing the model predictions with the measured values. Through a process known as ‘back propagation of error’, the error gradient is determined with respect to each node. The model parameters are then incrementally updated based on the error gradients, in an iterative process, until the model prediction resembles the measured output, as determined by a specified loss function. Common loss functions include mean squared error (MSE), or root mean square error (RMSE), though other options such as mean absolute deviation (MAD) are also possible. The model weights are updated via a mini-batch stochastic gradient descent as:

(3) $$W_{t+1}=W_{t}-\frac{\alpha }{b}\sum_{i=1}^{b}\nabla Q_{i}\left(W_{t}\right)\;$$

where $\nabla Q_{i}\left(W_{t}\right)$ is the gradient of the loss function with respect to the weight parameters, *α*, is a step size or ‘learning rate’, and *b* is the number of data points in each batch. 

An important limitation of the MLP for our context of groundwater modelling is that it does not naturally reflect the fact that the various observations are measured sequentially in time. Instead, each set of input/output pairs is treated independently. An advantage of this is that the MLP can still apply even if the outcomes are measured at irregular time points. Furthermore, some time linkage can be incorporated manually. For example, lagged values of predictor and outcome variables can be included as additional predictors. This means that the input data *X_t_* used to predict the outcome *Y_t_* at time *t* can also include information on *X* and *Y* values from times *(t* − 1), *(t* − 2) *…* and so on. Of course, this strategy will not work easily in settings where the time series has been measured sporadically. 

Time is incorporated into the local MLP models in this study both through the inclusion of lagged predictors, and through the addition of day-of-study and month-within-year variables to indicate overall time and seasons. As explained in Section 2, the extractions data are available only on an annualised basis, and the measured levels are assumed to be constant throughout the year. By including the month-within-year variable along with extractions, the model has the flexibility to create an interaction that allows the creation of an annual dip and recovery, with the depth of the dip dependent on the total number of extractions for that year. More detail can be found in [10], along with some discussion about how MLPs compare and contrast with more classical statistical methods. While such an approach may sometimes work reasonably well, the literature in recent years has moved towards more sophisticated ways to extend neural networks to apply specifically for time series prediction. 

### 3.3. Multi-Well Analyses

In this sub-section, we provide some details about the three different strategies that we use for multi-well analysis. The first one is based on the use of self-organizing maps coupled with LSTMs to create partitioned models, the second is based on the use of a global MLP model, and the third uses the global time series model DeepAR.

#### 3.3.1. Self-Organising Map (SOM)

The self-organising map (SOM, [22]) is an unsupervised neural network used for clustering, dimension reduction, pattern extraction, and data visualisation. The algorithm is inherently resilient to missing data, which frequently appear in monitoring data sets, due to differing measurement intervals and time series lengths. This attribute makes the SOM a popular choice for use in environmental analyses.

The map, or network, consists of a set of interconnected nodes in a grid format. A vector of dimension equal to the input data dimension is associated with each map node. Training the network involves an iterative process of dividing the input data into clusters that are nearest to each map node, relocating the map nodes to be nearer to their associated data, whilst maintaining the grid links, and then reassigning data to the clusters. This is done by comparing each item of data, $x_{i}$, to the map vectors, $m_{j}$ (where *j* = 1: M and M is the number of nodes) and determining the nearest map node, $m_{c}$, such that:

$$\left|x_{i}-m_{c}\right|=min_{j}\left\{\left|x_{i}-m_{j}\right|\right\}.$$

The node locations are then updated as:

$$m_{j}\left(t+1\right)=\frac{\sum_{i=1}^{N}h_{ij}\left(t\right)\;x_{i}}{\sum_{i=1}^{N}h_{ij}\left(t\right)}\;$$

where $h_{ij}$ comes from a neighbourhood function, indicating the influence of data item $x_{i}$ on the updating of node $m_{j}$ as follows:

$$h_{ij}\left(t\right)=exp\left(\frac{-{\left(m_{c}-m_{i}\right)}^{2}}{2\sigma^{2}\left(t\right)}\right)\;$$

where σ is the radius of the neighbourhood kernel, decreasing as a function of *t*. This continues until there is little change occurring with each update, and all data are associated with its nearest map node. At this point, the node vectors are considered representative of the data items associated with them, and these vectors indicate the most prevalent patterns in the data set.

Through this process, the most common patterns among the groundwater time series are determined and visualised by the SOM. The time series are placed into groups sharing similar temporal patterns over the timeline of the study. As the SOM is unsupervised, this step focuses only on the groundwater level data and does not incorporate any information from the predictor variables—climate, surface flows, and extractions are not considered when determining the clusters. For more information on the SOM algorithm, please see [23], and for applications to water resources, please see [24].

#### 3.3.2. Long Short-Term Memory Algorithm (LSTM)

A family of neural networks developed specifically for time series prediction, recurrent neural networks (RNN), incorporate the time domain directly into the network architecture. These models process the observations of a time series sequentially. In RNNs, each model output depends on all previous inputs, and the same set of weights is shared between all timesteps. A basic RNN, the simple Elman network [25], includes values of the hidden state at time (*t* − 1) in determining the hidden states at time *t*. Therefore, the prediction at time *t* depends not only on the predictors at time *t*, but also on what was happening at previous time points. The basic RNN can be represented as:

$$h_{t}=f\left(W_{ih}x_{t}+W_{hh}h_{t-1}+b_{h}\right)\;y_{t}=g\left(W_{ho}h_{t}+b_{o}\right)$$

where $W_{ih}$, $W_{hh}$, and $W_{ho}$ are the weight matrices between layers (input-to-hidden, hidden-to-hidden, and hidden-to-output), $b_{h}$ and $b_{o}$ are the biases, f(·) is the hidden layer activation function and g(·) is the output layer activation. With growing data sets and network sizes, these basic RNN models can suffer from issues during training due to vanishing or exploding gradients, meaning that the training signal becomes exponentially small or large as it travels through the network.

These issues have been overcome with the LSTM, an extended form of the RNN that allows for much longer time series to be analysed than could be previously. The LSTM solves the problem of the vanishing gradient through the incorporation of three ‘gates’ (the input, forget, and output gates) and memory cells into the network structure. These gates are in the form of additional nodes that regulate the flow of information in the network, allowing some information to be retained over long time periods, while other information is discarded. A sigmoid function at each gate regulates the flow by a value between 0 (forget everything) and 1 (retain everything). The memory cells are responsible for storing useful information from the past, enabling the model to learn the short-term as well as long-term dependent structure of the time series. The input, forget, and output gates (for the *m*^th^ memory cell) are modelled as:

$$i_{tm}=\sigma \left(\alpha_{0mi}+\alpha_{1mi}^{T}X_{t}+\alpha_{2mi}H_{t-1}\right)\;f_{tm}=\sigma \left(\alpha_{0mf}+\alpha_{1mf}^{T}X_{t}+\alpha_{2mf}H_{t-1}\right)\;o_{tm}=\sigma \left(\alpha_{0mo}+\alpha_{1mo}^{T}X_{t}+\alpha_{2mo}H_{t-1}\right)$$

where σ is the logistic sigmoid function and the $\alpha_{.}$ are matrices of learnable parameters for each gate, producing an output between 0 and 1.

The overall mathematical structure of the LSTM is a more complex version of the RNN model described above, involving updating the state of the memory cell based on the previous system state and the current input, with regulation by the input and forget gates. Using this updated system state, the memory cell output is calculated based on the output activation and output gate. The output gate regulates the flow of information from the cell state to the hidden layer. For a detailed description of the LSTM algorithm, refer to [26] or [27].

As the LSTM is recurrent in nature—that is, it cycles over the successive observations in sequence—lagged values of the predictors do not need to be entered as separate variables in the way that they are in the MLP. This algorithm assumes equal temporal spacing between the observations as it cycles through them sequentially, making it difficult to fit the LSTM to time series containing missing data, or with irregular measurement frequencies. LSTMs are able to incorporate external forcing data while determining multi-scale temporal dependencies. The LSTM is widely used for natural language processing, sentiment analysis, etc., where context is key. 

#### 3.3.3. Global MLP Model

The global MLP is created by fitting a single MLP to the data from all of the monitoring wells at once. As with any MLP, this is a static, supervised model that can do one-step-ahead forecasting or recursive forecasting. 

The input data consist of the time series from all the wells stacked into a single matrix. A well ID column is added to distinguish between observations that otherwise have the same predictors. Each row corresponds to information about a particular water level measurement. The columns of the dataset indicate: date, measured water level, well ID, and relevant predictors or features on that day (rainfall, streamflow, extractions, etc.). Additional columns include lagged values of rain and streamflow measurements. The outcome of interest, or target variable *Y*, is the water level, even though the water level is measured at numerous different wells. As with the local MLPs, time is incorporated into the global MLP model, both through the inclusion of lagged predictors and through the addition of day-of-study and month-within-year variables to indicate overall time and seasons. By including the well ID as a predictor, the global MLP can potentially create interactions between the environmental predictors and the individual wells, since rain and surface flows may have a different impact on different wells. 

An advantage of this global MLP analysis is that it does not matter if there are gaps in the dates on which water levels are measured, so long as all the predictor variables are available. In principle, an additional time series element could be introduced by adding columns to the dataset that correspond to lagged values of the water levels. While it may be appealing to consider doing this, a disadvantage is that the analysis will no longer work if there are gaps in the times at which water levels are measured. 

#### 3.3.4. Global Time Series Model (DeepAR)

An algorithm for fitting a global LSTM-based model to multiple time series, DeepAR [15] uses a scalable, supervised, deep-learning, recurrent neural network framework. Figure 3 from [15] illustrates the structure of the DeepAR model (note that they use *z* instead of *Y* to represent the outcome). The predicted value for the *i*th time series at time *t* depends on the value of the hidden nodes at time *t*, the value of the hidden nodes at time (*t* − 1), and the value of the outcome at time (*t* − 1). Note that this last feature brings in an autocorrelation element and is part of what distinguishes the DeepAR algorithm from a standard LSTM. 

In contrast to more traditional LSTM analyses, the DeepAR algorithm works by estimating the parameters of a probability distribution (rather than simply computing the predictions themselves), and then the probability distribution is sampled to produce predictions of the future. Another difference is that the DeepAR model has multiple target time series (in our case study, all of the measured groundwater levels). The model uses the water level at previous time steps as an input at each time step in addition to exogenous predictors. Though this information is available during the training phase, during prediction, the model recycles the predictions as inputs for the next time step. The DeepAR algorithm assumes that the same LSTM applies to all of the data, and then uses lagged values from the individual time series for predictions. The model is global in the sense that the same model is used for all the time series, with only differences in the input features (the *X*’s), as well as the past history of the series, driving differences between the predicted values for each series. Note that, as with the global MLP models discussed earlier, the identity of each time series can be included as an additional predictor, allowing the possibility of interactions being included in the prediction model. It is able to build in seasonality if needed through an automatic monthly indicator.

An added benefit of the DeepAR algorithm over the traditional LSTM (in addition to facilitating multiple time series predictions) is that it provides probabilistic predictions as well as point forecasts by combining the feature engineering capabilities of the LSTM with the probability forecasting capabilities of autoregressive models such as ARIMA.

DeepAR requires the specification of various hyperparameters such as the context length (how far into the past the model looks back), the prediction interval (how far into the future the prediction is to be made), as well as the usual hyperparameters required for the MLPs, including the number of network layers, number of nodes per layer, number of training epochs, batch size, and dropout rate. The context length has a significant effect on DeepAR predictions, and we found better prediction results when context length was similar to prediction length.

### 3.4. Summary of Methods

Table 1 summarises the analysis strategies that we will apply to the groundwater time series data from the Namoi valley. The predictors and response data differ between models, as do the levels of data aggregation (daily or monthly), and these choices are explained in the next section. Predictors include measured values of rainfall, evapotranspiration, surface flows, extractions (along with their lagged values), and time indicators. 

All predictors and target variables were pre-processed by scaling to the range [0, 1] before use in the models. This allows for comparisons to be made between time series with groundwater level changes of differing magnitudes. As these differences in scale are related to the unknown horizontal extent of each aquifer, they are not relevant to this study of time series patterns. Scaling the predictors as well as the groundwater levels is an important step to ensure a relatively similar order of magnitude of the neural network weights within a model.

The accuracy of the various methods is characterised in terms of prediction performance on data that were not seen during the training process. Evaluations of the models are given using the root mean square error (RMSE) metric. RMSE measures the square root of the average of the squared differences between the observed and predicted values, or more simply, the standard deviation of the prediction errors. 

### 3.5. Software

All analyses in this study are performed with open-source software. The MLPs and LSTMs use the ‘keras’ package [28] in R version 3.6.3 [29]. The SOMs are run in R using the ‘kohonen’ package [30]. The DeepAR models are run in Python using the ‘deepar’ package from GluonTS [31], a toolkit for probabilistic time series modelling. GluonTS is an interface of the Apache MXNet library for deep learning. DeepAR is also available as part of Amazon Web Service’s Sagemaker program [32].

## 4. Modelling Setup and Results

### 4.1. Single Well Analyses

Individual MLP models were created for all time series with more than 2000 daily observations, which are those corresponding to the telemetered wells. This is a subset of 11 time series, each having between 2589 and 4113 daily measurements. When converted to monthly time series, these 11 telemetered wells have between 230 and 389 data points each. Two MLPs are run for each; one using daily data and another using monthly data. Measured regional rainfall, evapotranspiration, surface flows, and extraction data are used to predict groundwater level at each time step. 

In order to incorporate time into these static models, two strategies are used. First, we include lagged predictor data (30 days lags of rainfall and surface flows when using daily data and 12 months lags when using monthly data) and secondly, day-of-study and month-of-year variables are added. The number of lagged inputs for the monthly models was determined by first running some exploratory generalized additive models (GAM) to identify the lag numbers that could explain the high degree of observed variability. For the daily models, the number of lags is capped at 30 to limit the number of predictor variables. Uneven gaps between the observation dates for the water levels are not an issue with the MLPs, since each observation contains all the lagged predictors within it.

Two-layer networks are used for the individual MLPs, with 32 and 16 nodes on the first and second layers, respectively. Overall, 70% of the data are used for training, with 15% each reserved for validation and prediction. The batch size is 32, meaning that the network weights are updated after each batch of 32 data points are seen by the model. The models are run for up to 200 epochs (an epoch is one run-through of the entire set of input data points) with early stopping if the prediction of the validation portion fails to improve over 20 consecutive epochs. Dropout is set at 15%, so that a random 15% of nodes are deactivated during each training epoch in order to prevent the overfitting of the network. For more information on the use of early stopping and dropout to prevent overfitting during neural network training, please see [26].

**Figure 7:**
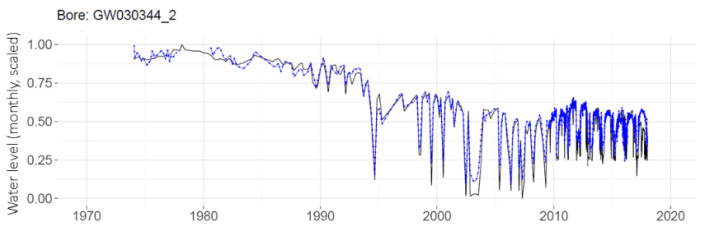
Individual well MLP daily predictions (blue, dashed) and observations (black). RMSE = 0.134.

The MLP prediction of daily water levels at one individual well is shown in Figure 7 compared with observed data. The predictions from the MLP (in dashed blue) can be seen to generally follow the observations (in black), and to capture the annual drawdown pattern of the latter portion of the time series. Root mean square error (RMSE) is reported for this and subsequent figures to indicate fit accuracy. Individual MLP water level predictions for the set of 11 telemetered wells are provided in Appendix A. 

**Figure 8:**
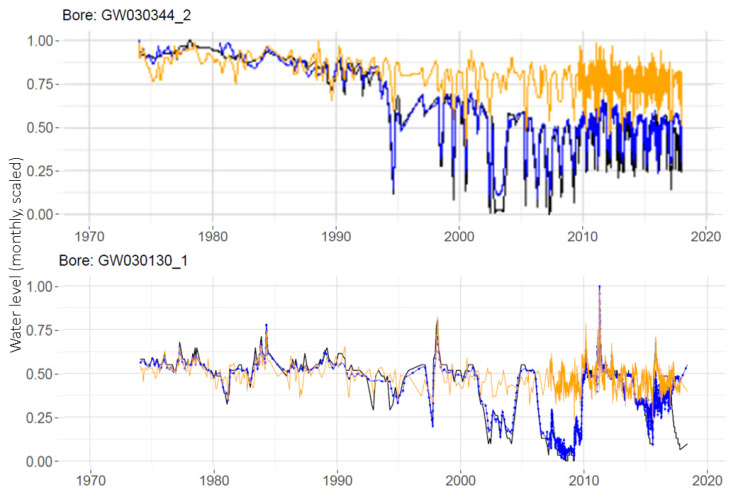
MLP predictions for two example wells (blue) over observed data (black), and predicted water level if extraction data are set to zero (orange).

The MLP results offer useful insight into the impact of climate and extractions on water levels. For example, the effects of extractions can be investigated by setting the extraction volumes predictor variable to zero in the prediction phase. Figure 8 shows the results of this for well GW03044_2 from Figure 7 and for a second well, GW03130_1. The orange lines show the predictions when extractions are set to zero. The figure suggests that extractions are explaining a good amount of the observed annual fluctuations seen in the data. It can be seen that the water levels for the well on the upper panel were predicted to be consistently higher over the last few decades if extractions had not been occurring, whereas the well on the lower panel appears able to recover to non-extraction levels in between times of considerably drawn-down water levels (for example, where the black measured data and blue ‘with-extractions’ prediction rebound to meet the orange ‘without-extractions’ prediction around 2012).

**Figure 9:**
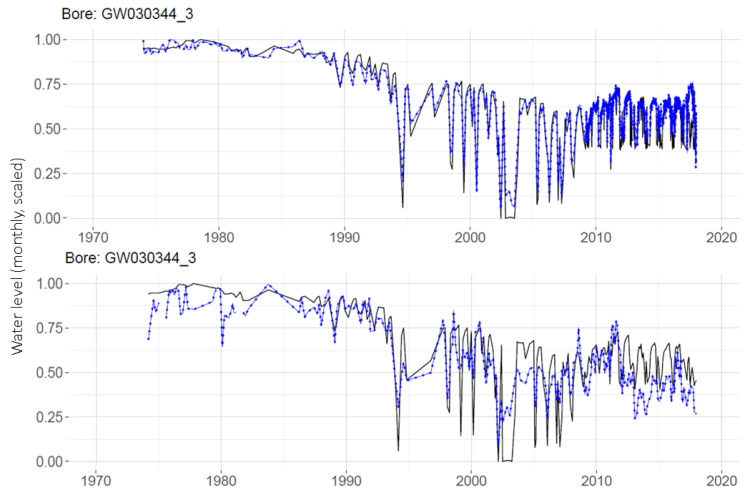
Comparison of MLP predictions (blue, dashed) over observed data (black) using daily data (upper panel) and monthly data (lower panel) for an individual well.

The MLPs are run with daily data sets and monthly aggregated data sets. A comparison of the daily and monthly results for one well is shown in Figure 9. It can be seen that the MLP with daily data (on the top panel) has the advantage of better prediction results in the latter years, since during this era, the measurements were collected on a daily basis after the station was telemetered. By aggregating this into monthly data, the benefit gained from the high number of training observations in this latter era is lost, as seen on the lower plot.

A significant drawback to using an MLP model with time series is that information is not naturally incorporated about the order of sequential observations. As recent information is often more valuable to predictions, this method is not ideal for time series analyses. Furthermore, the MLPs developed in our setting cannot be used to investigate the effects of predictors into the future, since they include an additional time variable, needed to explain the overall temporal trend. By including these time variables, the fitted models are operating somewhat similarly to how a GAM operates when fitting a flexible function of time to the dataset. The temporal trend is fit specifically to the available data and may not generalise well for the future for forecasting. Lastly, the use of individual models means that no sharing of information can take place between the sites.

### 4.2. Multi-Well Analyses

#### 4.2.1. Partitioned Analysis with SOM and LSTMs

The self-organising map algorithm is used to explore the relationships among the multiple time series and to group them into sets of smaller clusters for use in the partitioned multi-well analysis. Then, LSTMs are fit to a representative time series from each subset (or cluster) obtained from the SOMs. Each LSTM is able to be used for the prediction of groundwater levels for any of the wells within its cluster. This work was published in [18] and is summarised here as a basis for the partitioned models of this project. 

##### Self-Organising Maps

For an initial exploration of the relationships between the multiple time series, the self-organising map algorithm is used to obtain: the common groundwater patterns in the region, clusters of time series with similar patterns, and visualisations of the historic groundwater patterns with information on the geographic relationships between them. 

**Figure 10:**
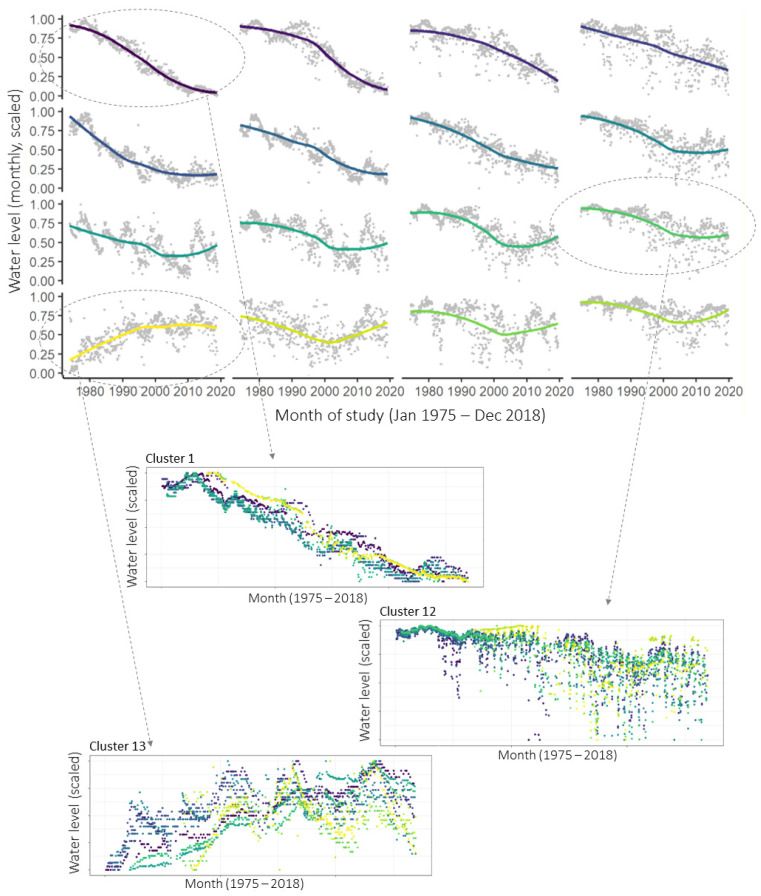
SOM clusters, identifying groups of wells with similar temporal patterns. The upper panel shows the 16 most prevalent groundwater level patterns found in the dataset. Coloured smoothers are added to indicate the predominant trend in each cluster, with similar colours indicating more similar patterns. The lower portion shows examples of measurements taken at wells in three of these clusters, with data points coloured by measurement well (there are 9, 11, and 10 wells allocated to clusters 1, 12 and 13 respectively). The colours in the lower panels are present to identify the measurements taken at one well from those at another and do not relate to the colours in the upper panel. Adapted with permission from ref. [18]. Copyright 2022 Elsevier.

When applied to the time series from wells in the Namoi basin, the SOM identified 16 clusters of time series that share similar temporal patterns over the timeline of the study. This indicates the 16 most prevalent water level patterns among the monitoring data. These 16 ‘representative’ patterns are shown in grey on the upper panel in Figure 10, with coloured smoothers indicating the overall trends in each cluster. The patterns are arranged automatically on the SOM output map so that nearby patterns are more similar than distant patterns. The coloured smoothers highlight some attributes of the predominant groundwater level patterns in the region. For example, all clusters except the one in the lower left corner had declining trends in 1974–2000, and clusters in the very top right continued to decline at the same or greater rate until 2018, whilst others lessened in rate of decline or had an upward trend after the mid-2000s.

Each well time series is matched to the pattern (and therefore becomes a member of the cluster) that it is most similar to. On the lower portion of Figure 10, the measured time series that make up three of the clusters are shown as examples, with dots corresponding to observations from the same measurement well being shown in the same colour. It can be seen that the nine wells allocated to Cluster 1 have steadily decreasing water levels over the length of the study. Water levels in the 11 wells at Cluster 12 are also generally decreasing, with annual drawdowns beginning to significantly increase in depth around the middle of the study period. The 10 wells at Node 13 show generally increasing water levels and similar multi-year fluctuations. 

**Figure 11:**
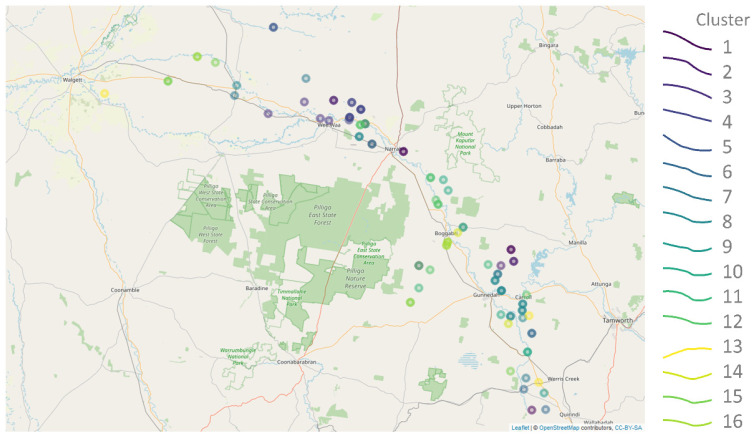
Visual analysis of historical data with the SOM. Well locations are coloured by the SOM cluster that is best matched by their historical time series (1974–2018). The time series’ general patterns for each colour are shown in the legend. Adapted with permission from [18]. Copyright 2022 Elsevier.

Figure 11 shows the location of various wells in the Namoi Valley, colour-coded according to their SOM cluster. It is interesting to note that, while some wells within the same cluster are located in close geographic proximity, many are not. This reflects the reality that the various wells are likely to be accessing different aquifers or separated by unknown underground discontinuities.

It is important to note that the SOM analysis is not useful for prediction, since it is purely empirical and exploratory. It focuses only on the groundwater level data and does not incorporate any information from the predictor variables—climate, surface flows, and extractions are not considered when determining the clusters. For a further analysis of the SOM results, please see [18].

The clusters found by the SOM can now be used in the LSTM portion of the partitioned multi-well analysis. The SOM has produced complete monthly data sets (with no missing data over the study period) in the form of the clusters’ representative time series, and these can be easily input into LSTMs in the next step.

##### LSTMs

The deep learning algorithm for time series, LSTM, is applied to the clusters’ representative time series to investigate the relationship between the groundwater levels and external predictors for each of the SOM clusters. As there are 16 SOM clusters, 16 LSTMs are produced at this stage. Predictor variables are now added into the analysis, including: rainfall and evapotranspiration, river flows and extraction data, and a month-of-year variable. All input data are monthly averages, and the representative time series from each SOM cluster is the target variable for each LSTM. 

LSTMs with two layers are used, with 50 nodes on the first and 25 nodes on the second layer. The first 80% of data are used for training, with 10% each for validation and testing. The LSTMs are run for up to 500 epochs with early stopping after 20 epochs, a batch size of 32, and a weight decay parameter (lambda = 0.00001) to regularise weight estimates and reduce overfitting. During training, the LSTM algorithm ‘looks back’ over the data for a specified number of time steps, cycling sequentially through the observations and retaining important information through time. The LSTMs are run with a variety of possible look-back lengths (3, 6, 8, 10, and 12 months) and the model providing the best overall match of model output with each cluster’s representative time series is chosen.

**Figure 12:**
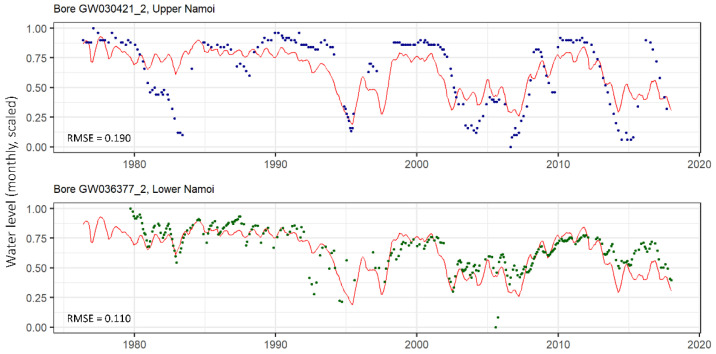
LSTM prediction (red) for one cluster shown with measurement data from an un-telemetered well in the Upper Namoi region (upper panel, blue dots), and an untelemetered well in the Lower Namoi region (lower panel, green dots), showing that the same LSTM prediction can apply to wells in different regions that are members of the same cluster. Adapted with permission from [18]. Copyright 2022 Elsevier.

A sample of LSTM results is shown in Figure 12 for one of the clusters (cluster 15). The LSTM model prediction (in red) is shown with the measured data (coloured dots) for two wells in this cluster. These are un-telemetered wells: one from the Upper Namoi region, and one from the Lower Namoi region, neither of which would have been able to be modelled individually with the LSTM due to the intermittent time series. Though these wells are located in different parts of the groundwater system, it can be seen that the groundwater levels at both are generally well predicted by the single LSTM. 

The partitioned models created with the SOM and LSTM combination are able to provide predictions for all of the groundwater time series, even those with very few observations. This is attributed to the SOM algorithm being able to match the wells with low numbers of observations into clusters that share similar temporal patterns, and then representing these wells in the LSTMs with the clusters’ representative time series. Neural networks in general need large datasets for training, and the LSTMs here are trained on the full representative time series, whilst allowing predictions to be made for the individual wells, regardless of the number of measurements at each well. Though this method has benefits, a drawback is that the same prediction is used for all wells within each cluster. 

#### 4.2.2. Global MLP

A global MLP model is produced that incorporates information from all the wells. This allows for model training with a much larger dataset and aims to provide insight into what is happening at a catchment-wide level. Data are aggregated to the monthly level for use in the global MLP in order to improve computational efficiency over the use of daily data. This aggregation applies not only to the groundwater measurements, but also to all the predictor variables and their lags. The size of this data set is 36,142 observations with 368 predictor variables, which includes 12 lags on all rain and streamflow gauge measurements (at monthly aggregations). 

**Figure 13:**
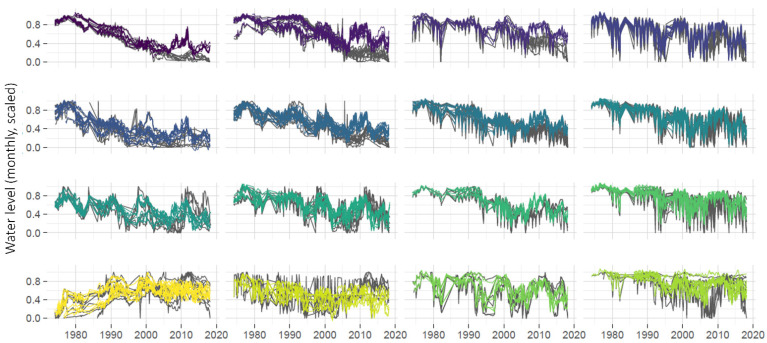
Global MLP groundwater level predictions for 165 time series (grouped and coloured here by SOM cluster as described in Figure 10) shown over observations (black). RMSE = 0.239.

The global MLP is fit using a two-layer network with 64 and 32 nodes on the first and second layers. All other hyperparameters are the same as for the individual MLPs, as described above. Figure 13 shows the results of fitting this global MLP, with results clustered purely for the sake of display according to the cluster groups identified in the SOM analysis. Global MLP predictions are indicated in the respective cluster colours, superimposed over the observed groundwater level measurements for the wells in each cluster (in black). Though the SOM cluster membership of each time series is not known to the global MLP, it can be seen that the model produces different patterns for the various clusters. 

**Figure 14:**
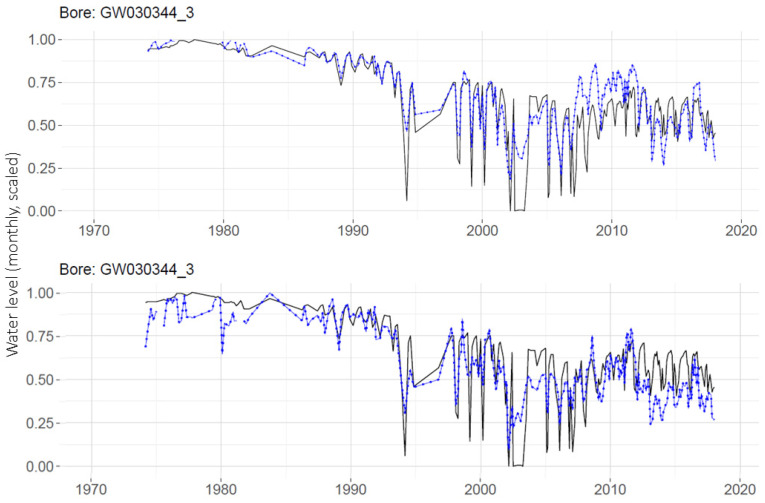
Comparison of monthly predictions from the global MLP (upper panel, RMSE = 0.114) and individual monthly MLP (lower panel, RMSE = 0.156) for the same well. Predictions are in dashed blue and observations in black.

Predictions produced by the global and individual MLPs are compared in Figure 14 for a single well, GW030344_3. Using the global MLP with monthly data, the prediction for this well (RMSE = 0.114) is an improvement on the prediction for this well made with an individual MLP (RMSE = 0.156). Note that the RMSEs reported here are over the testing portion only.

**Figure 15:**
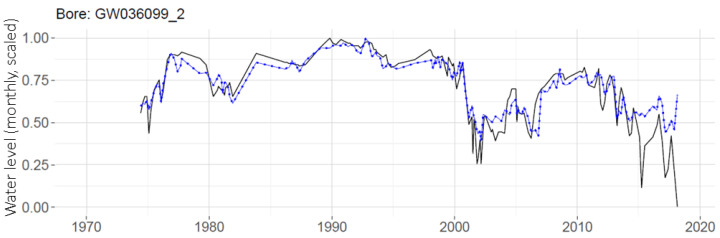
Prediction on an untelemetered well using global MLP (blue, dashed) over measured data (black). RMSE = 0.195.

Though the use of global MLP indicates some improvement for predictions on a telemetered well as shown in Figure 14, the real advantage of global MLP is its ability to provide predictions for non-telemetered wells (those with few data points) for which individual MLPs were not made. One such well, GW036099_2, has only 165 measurements over the 45-year study period. A prediction made for this well with the global MLP is shown in Figure 15.

As the global model used here is based on the basic MLP algorithm, it also does not fully exploit the time series nature of the data, as was the case with the local MLPs. Below, we apply another global model. This one is based on the LSTM which, as discussed above, specifically incorporates the sequential nature of the data into the algorithm. 

#### 4.2.3. DeepAR (Global LSTM)

A DeepAR model is another form of global model, and is therefore trained on all of the time series. This model uses the LSTM algorithm to analyse sequential data and is able to provide probabilistic predictions. The structure of this model differs from the global MLP in that rather than a single target variable of ‘measured water level’, all of the groundwater monitoring wells’ time series become separate target variables, giving 165 target variables.

We trained a two-layer DeepAR model with 32 nodes on each layer for 200 epochs, with a dropout rate of 10%. The context length is five years, and the prediction length is five years. The input variables are separated into categorical predictors (which are static for each time series) and dynamical predictors (which change over time for each time series). One categorical predictor, the well ID, is used in our model. The dynamical predictors are rain, evapotranspiration, surface flow, and extractions, as in the MLPs. Twelve months of rain lags are included in the input data set. In addition, the year-of-study is included as a dynamical predictor.

**Figure 16:**
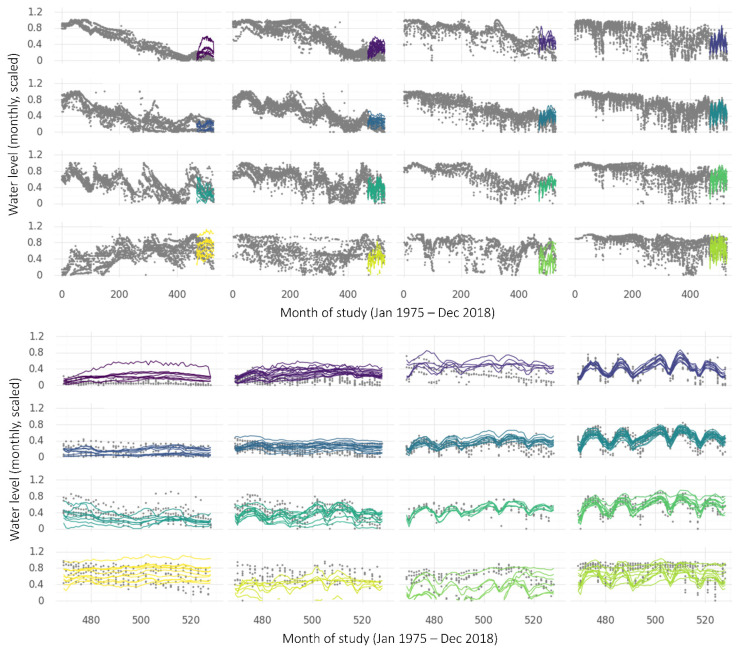
DeepAR predictions for the last 5 years of each of the 165 time series arranged and coloured by SOM cluster membership (as described in Figure 10) with observed data in grey. Top row: entire study duration (1974–2018); Lower row: final 5 years of study (January 2014–December 2018). RMSE = 0.209.

The DeepAR results, shown in Figure 16, are plotted by the SOM cluster membership of each time series (as with the global MLP, this is only for ease of visualising the multiple time series; cluster membership is not used in training the model). Actual bore measurements are plotted in grey, but predicted values are plotted using the colour key shown in Figure 11. The upper panel shows the predictions for the last five years of the study (RMSE = 0.209) coloured by the SOM cluster, overlaid on the observed data for the entire study period. The lower panel is zoomed in on the five years of prediction (i.e., the final five years of the study).

**Figure 17:**
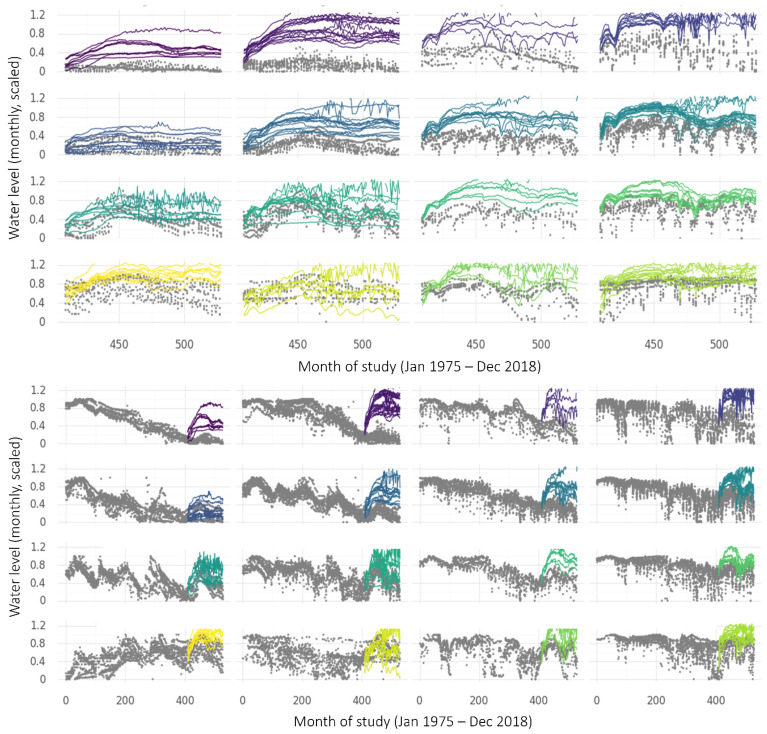
DeepAR predictions for the last 10 years of the study period, arranged and coloured by SOM cluster membership (as described in Figure 10) with observed data in grey (RMSE = 0.477).

Whilst the DeepAR predictions follow the observations relatively well over the 5-year testing period, the results were less promising when trying to predict further into the future. Attempting 10-year predictions (as shown in Figure 17) produced very unsatisfactory results (RMSE = 0.477). Model setup and input data were the same as for the 5-year predictions, with the exception of doubling the prediction length.

### 4.3. Summary of Results

To evaluate the models, we compare the prediction performance on the testing portion of each time series using the RMSE metric. Results are listed in Table 2. For the individual or partitioned models, the RMSEs are weighted by the number of measurements in each time series or cluster and summed over the individual time series (or clusters), such that $RMSE_{weighted}=\sum_{i}\frac{n_{i}}{N}RMSE_{i}$ where i denotes the individual time series or cluster, $n_{i}$ is the number of measurements in the time series or cluster, and N is the total number of measurements in the set of time series. 

## 5. Discussion

An exploration has been made into the benefits of using global models to incorporate time series information from multiple time series, rather than analysing data from single wells one at a time. A number of advantages exist within this global modelling strategy: the input datasets are larger, which generally leads to the improved performance of machine learning approaches by providing more training examples; and by exploiting common patterns across the different wells or subsets of wells, relevant patterns can be captured in a more efficient and parsimonious manner. In terms of prediction accuracy, global time series neural network approaches have shown impressively good properties in practice. Results from the recent time series prediction M4 and M5 [33] competitions on 10,000 s of time series showed overall domination by RNN-based deep learning global modelling strategies.

In this study of groundwater monitoring data, both the local models and the global models did well in terms of the overall prediction RMSE. When it came to predictions for individual wells, we found that, in some cases, the global models outperformed the local models, and in other cases, the reverse was true. While both the local and the global models were moderately successful in explaining the observed strong annual patterns seen for some of the wells, neither were completely ideal for all of the wells. This may be a result of the differing responses to the predictor variables or the differing number of data observations at the various wells. The limitations of the available data on extractions may also be a major contributing factor. 

When it came to prediction of the future, the methods struggled when we tried to predict too far ahead. In particular, while results could be considered satisfactory in terms of RMSE when predicting the relatively short-term (<5 years), the results deteriorated substantially when trying to predict for a longer period of 10 years. This phenomenon is quite typical for time series prediction, which works by exploiting the autocorrelation structure inherent in the data to predict the future. For a reliable long-term prediction, the best strategy is unquestionably to make sure that the analysis has access to the right predictors or features that can explain the observed patterns. 

Although there are limitations in terms of how reliably the data can be projected into the far future, the results demonstrate the usefulness of exploring “what if” scenarios such as setting extractions to zero. These explorations could be expanded to see how the predictions might look under scenarios, such as multiple successive years of very low or very high rainfall. 

In terms of identifying a best modelling strategy for this set of environmental monitoring time series, no single approach was found to be uniformly best across all time series. The choice of best strategy is complicated by the fact that differences between the various methods can be subtle, and there are many trade-offs in terms of prediction outcome and ease of working with the data at hand. The choice of the best strategy depends strongly on the context; the best strategy for short-term prediction is likely to be quite different from the best strategy for long-term prediction. There are also differences in how successfully the various approaches can be adapted to handle limitations in the available data. Whilst it is straightforward to adapt the MLP-type models to handle time series with missing data points or time series measured at sporadic timepoints, it is difficult to do this for the LSTM model. There are also some technical differences between the various methods in terms of the software available for implementation. 

This particular analysis was challenged by the variability in the frequency of groundwater level data collection. Although data collection goes back to the early 1970s for many wells, the earlier measurements were based on manual collection every 6 weeks or so. Starting in the early 2000s, some of the wells were fitted with automatic telemeters that provided continuous monitoring. As a result of this variability in measurement frequency and timing, it was virtually impossible to fit LSTM-type models to the daily data. This can be circumvented by aggregating to monthly data, however this aggregation potentially results in some loss of predictive power. Based on our local MLP analyses on individual wells, we found that RMSE was indeed better when daily data were used. Another strategy may be to employ approaches to infill the missing data, but we have not done this, as this study was aimed at determining what strategies could be used with the raw, sporadic data, as measured. 

Strong temporal patterns were found for many of the wells, as seen with the MLP modelling on the individual telemetered wells and with the SOM clustering analysis. As discussed earlier, we were able to develop models that fit the observed data by including appropriate time terms in our models, along with the observed climate and extraction predictors. However, while such models may explain observed data very well, they cannot be reliably used to predict far outside the range of observable data. Similarly, we found that models incorporating appropriate autoregressive terms could do a good job in terms of short-term predictions. For long-term prediction, however, it is critical to develop models that have a rich enough set of reliable predictors to explain the time trends. The importance of exogenous variables for improving time series forecasting accuracy was also listed as one of the main takeaways from the M5 competition [33].

**Figure 18:**
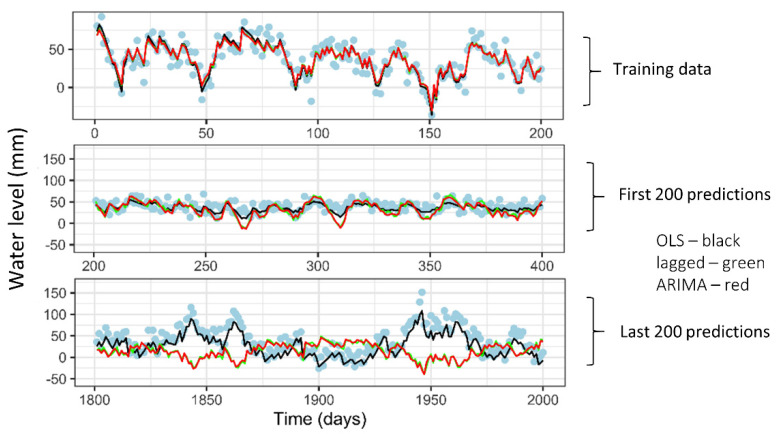
Top panel: Observed data (blue dots) for first 200 datapoints, along with fitted values from the linear regression models. Middle panel shows observed data and predicted values from the two linear models, as well as the ARIMA model for the short-term prediction scenario (days 201–400). Bottom panel shows the observed data and predicted values from the two linear models as well as the ARIMA model for the long-term prediction scenario (days 1800–2000). In all panels, OLS predictions are shown in black, linear model with lags in green, and ARIMA in red.

To further explore the importance of the long-term prediction of ensuring the right predictors are captured, a small computer simulation was conducted. Specifically, we generated a time series containing 2000 datapoints. The first 200 points were used for model fitting, and then the models were evaluated for how well they could predict (a) the next 200 points and (b) the final 200 points. Scenario a) could be considered an example of short-term prediction, whilst scenario b) could represent a long-term prediction. The data-generating model included a predictor that mimicked a rain variable, as well as some seasonal patterns and a long-term trend. The models that were fit included a linear regression model (ordinary least squares or OLS) that included the correct predictors, a linear model that did not include all the correct predictors, but included a lagged outcome variable, and finally, a classic time series model (ARIMA) which could exploit the autocorrelation structure in the data. Figure 18 provides the results, with the top panel showing the data used for model fitting (blue dots), along with the fitted values from the two linear models. The black line corresponding to fitted values from the linear model is virtually identical to that corresponding to the fit from the linear model with lags (green line), as well as the fit from the ARIMA model (red line). The same can be seen in the middle panel which shows the predictions for the short-term scenario (predicting the next 200 timepoints). However, the bottom panel showing the long-term prediction results shows that, while the linear model still does an excellent job, the two “time series” models (linear model with lags as well as ARIMA) both do very poorly. This is a fairly simplistic exercise based on a simple simulation, but it underscores the reality that classic time series methodologies cannot be relied on for long-term predictions. A much better strategy is to make sure that all of the right features or predictors have been identified and, where possible, used to predict the future.

While it is beyond the scope of this paper to discuss in more detail, it is interesting to note that some time series analysis programs provide error bands around their predictions, which shows the uncertainty of prediction of the future (see, for example, Figure 1, which includes such bands in the ARIMA results fitted to the Richmond River catchment). Typically, these prediction bands quickly become very wide, illustrating the phenomenon well. DeepAR is in fact one of the programs that provides prediction intervals as an option. Whilst not shown, we did generate some prediction bands for the DeepAR models and, as expected, they became very wide quite quickly. The other programs used here (MLPs and LSTMs via Keras in R) did not provide the option to generate prediction intervals. It is a well-known phenomenon that prediction intervals based on time-series methodologies tend to become very wide as they predict further and further into the future. This is because time series predictions of the immediate, short-term future are able to exploit the autocorrelation structure inherent in the data. Longer term, however, it is only via reliable endogenous predictors that one is able to obtain an accurate prediction.

There are a number of analytical strategies that could potentially be explored to improve the predictive power of our models. Transfer learning, for example, involves pre-training global models on all of the data, and then further fine-tuning the models for a small number of epochs to get a better fit for each specific well. Moreover, ref. [34] applied such a strategy in the context of predicting runoff, and the fine-tuning step was found to improve runoff prediction over just using the global model as-is. The authors also found that the process works even if the data used in the global model is quite inhomogeneous. Ref. [35] used transfer learning with models developed for a set of monitored lakes to make predictions at unmonitored lakes, and found it to be a powerful technique for transferring knowledge learnt in areas with sufficient data to areas with scarce or inadequate data. Our analysis had a catchment-specific focus. The literature contains examples where similar kinds of statistical and machine learning methods had been used to model data based on multiple catchments. Ref. [17], for example, developed models that applied to the entire state of Victoria, Australia. The authors did not have access to extractions data, but used remote sensing data collected through the GRACE satellite program [36]. The use of such remote sensing data in place of the annual extractions data may be a useful option for further work on our study, as well as considering other data sources related to land use. 

Exploratory analyses using some simple GAM models suggested that there were strong interaction effects between some of the variables and the time indicators. Such a phenomenon could well be expected, in the sense that some of the observed predictors may be effectively explaining the rate of decline or change in groundwater levels. The LSTM models are flexible enough to capture such effects. However, it is very difficult to tell whether or not the models are adequately explaining how levels change over time in a way that can be reliably projected into the future for forecasting. There are some analytical strategies that could potentially be explored to address this issue. One option is to apply some of the recently developed strategies related to explainable AI (see [37]). Another worthwhile angle would be to explore state space modelling ideas (see classic text by [38]). Such models have been developed and explored extensively in the time series literature and involve making the assumption of the existence of a latent time trend in the data, however they have limitations in terms of how predictors can be incorporated. An ideal situation may be to combine ideas of neural network modelling with state–space modelling, including the possibility that covariates can impact the rate of change of the process being modelled.

## 6. Conclusions

Local, partitioned, and global algorithms were investigated here for making predictions based on monitoring time series data for groundwater levels, and each was found to have benefits and drawbacks depending on the context and characteristics of the monitoring data. 

The local (individual) MLP models were able to incorporate the sporadic datasets and provide quick and easy predictions, though it was necessary to add temporal features manually. These individual models were restricted to the few groundwater wells that had automatic telemeters installed, as single wells with manual measurements do not have enough data points for the MLPs. The results of the telemetered wells showed relatively good predictive accuracy with the local MLPs. 

The partitioned models, using the SOM algorithm to partition the data sets into clusters that could be modelled with LSTMs, were also able to provide acceptable predictions. The benefit of these partitioned models is that they are able to provide predictions for the entire set of time series, even those with very few measurements. A drawback of this method is that the same LSTM prediction is given for a number of individual wells, and therefore may result in a loss of accuracy in the prediction of groundwater levels at telemetered wells for which the individual models worked well.

The global models were a good solution for increasing the size of the data set, for sharing information catchment-wide, and again for providing predictions at wells with few data points. The DeepAR algorithm offers a powerful modelling framework to fit a global LSTM model to the time series from all the wells. The framework has a number of advantages, including the ability to create needed interactions and nonlinearities, however a primary emphasis of DeepAR is the incorporation of autoregressive terms; this is the ‘AR’ part of DeepAR. We have discussed how these are very helpful for predictions in the short term, but not for long-term predictions. 

Overall, it was determined that, whilst these methods are able to do a satisfactory job of modelling groundwater levels with the use of appropriate covariates, it is nonetheless not straightforward to use them for the prediction of the future. Our analyses of the Namoi catchment data suggest that, even though data related to climate, extractions, and streamflow/dam releases can explain a relatively high proportion of the observed trends in the regional groundwater levels, it appears that some residual temporal effects remain, even after these variables have been accounted for. Model fit can be improved through the inclusion of time effects into the modelling process, and the inclusion of autoregressive terms can also help to “soak up” temporal effects. However, for the purpose of projecting further into the future, it is important to have models that can explain temporal effects through accurately measured features. Additionally, it follows that the predictions of these features into the future (e.g., future expected rainfall or future extractions) must also be accurate if they are to be relied on as predictors in the groundwater level models.

An interesting outcome of this study is the potential for the use of these methods for a relatively easy analysis of ‘what-if’ scenarios, as shown in Figure 8, where the groundwater levels were estimated for a fictitious scenario in which no pumping had occurred in the catchment. The ability to easily add and remove potential predictors from an analysis and compare the predictions is a strong drawcard for these methods.

There are a number of possible directions for future work. As discussed above, improved strategies for reliable measurement of extractions would likely improve model fits. It is intriguing to consider the possibility of using other data sources such as satellite data to capture land use and changes in water storage. We also believe that there are further analytical directions that could be explored. In particular, we recommend exploring the use of state–space modelling combined with a deep learning framework. This would require the development of new statistical methods, as well as software engineering. It would also be useful to develop a modelling framework that incorporated a global LSTM structure like DeepAR, but which allowed the option to turn off the autoregressive component, or allowed for the number of AR terms to be delinked from the “lookback” component of DeepAR. Such a modified version of DeepAR would allow for analyses that focus on explaining the observed trends in the data, without simply “soaking up” the effects with AR terms.

## Figures and Tables

**Table 1:** Summary of algorithms and input data used in the study. Frequencies of observations used in the models are denoted by D = daily, M = monthly or A = annual frequency.

Algorithm	Purpose	Response Data (Groundwater Levels)	Predictors
**MLP (individual)**	Input/output predictions	Individual time series	Rainfall (+30D, 12M lags) (D, M), Evapotranspiration (D, M), Surface flows (+30D, 12M lags) (D, M), Extractions (A), Day-of-study, Month-of-year
**SOM**	Unsupervised clustering and dimension reduction	Clusters of time series	All of the groundwater time series (M)
**LSTM**	Deep learning time series predictions	Representative time series from each cluster	Rainfall (M), Evapotranspiration (M), Surface flows (M), Extractions (A), Month-of-year, (Lookback varies by cluster)
**Global MLP**	Input/output predictions for multiple monitoring data sets	All time series	Rainfall (+12M lags) (M), Evapotranspiration (M), Surface flows (+12M lags) (M), Extractions (A), Well ID, Month-of-study, Month-of-year
**DeepAR**	Multiple time series probabilistic predictions	All time series	Rainfall (+12M lags) (M), Evapotranspiration (M), Surface flows (M), Extractions (A), Well ID, Year-of-study (60-month context length)

**Table 2:** Summary of results in terms of root mean square error (RMSE) for the various modelling strategies employed.

Method	Aggregation	Number of Series	Number of Data Points	Lagged Predictors	Average RMSE **
**Individual MLP**	Monthly	11 *	299 (mean per series)	12 months rain lags	0.195
				12 months rain and surface flow lags	0.165
	Daily	11 *	3212 (mean per series)	30 days rain lags	0.163
				30 days rain and surface flow lags	0.153
**Global MLP**	Monthly	165	36,142	12 months rain and surface flow lags	0.239
**Partitioned LSTMs**	Monthly	16 ^	540 (per cluster)	Various lookback lengths	0.198
**DeepAR (5 year)**	Monthly	165	24,839 (47 predictors, 528 months)	12 months rain lags	0.209

* Individual MLP models are run for telemetered stations only. ^ Partitioned LSTMs are run for each of the 16 clusters. ** Average root mean square error, weighted by number of measurements for each well or cluster.

**Figure A1:**
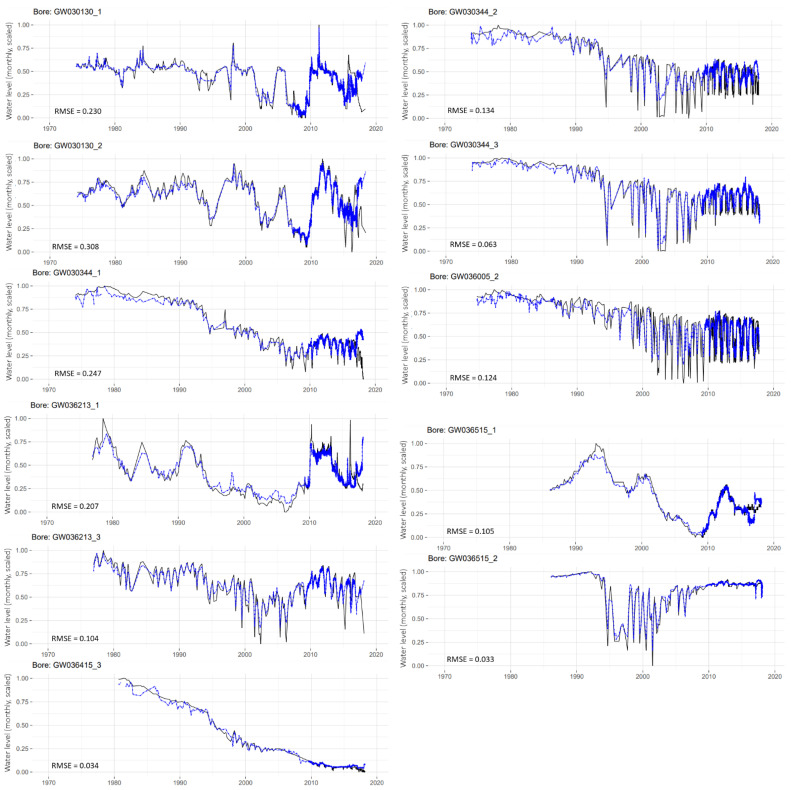
MLP predictions for the set of telemetered wells.

## Data Availability

Data used in this study are publicly available from WaterNSW (bore measurements, flow rates—see https://www.waternsw.com.au/waterinsights/real-time-data) or from the Queensland Government (infilled rainfall measurements—see https://www.longpaddock.qld.gov.au/silo/). The analyses in this paper were based on data accessed on 1 March 2020.

## Appendix A

MLP predictions are shown for the 11 telemetered stations using daily data. Observations are in black and predictions in blue.
